# Primary breast non-Hodgkin’s lymphoma in a 14-year-old girl: a case report

**DOI:** 10.1186/s40792-020-00850-9

**Published:** 2020-04-29

**Authors:** Yumiko Ishizuka, Yoshiya Horimoto, Junya Fujimura, Kozue Ogata, Fumi Murakami, Hiroko Onagi, Atsushi Arakawa, Mitsue Saito

**Affiliations:** 1 Department of Breast Oncology, Juntendo University School of Medicine, 2-1-1 Hongo, Bunkyo-ku, Tokyo, 113-0033 Japan; 2 Department of Human Pathology, Juntendo University School of Medicine, 2-1-1 Hongo, Bunkyo-ku, Tokyo, 113-0033 Japan; 3 Department of Pediatrics, Juntendo University School of Medicine, 2-1-1 Hongo, Bunkyo-ku, Tokyo, 113-0033 Japan

**Keywords:** Primary breast lymphoma, B-lymphoblastic Lymphoma, Children, Surgery

## Abstract

**Background:**

Primary breast lymphoma is rare. Occurrence rates of malignant breast tumors in children are also quite low. We herein report a B-lymphoblastic lymphoma of the breast arisen in an adolescent girl. To the best of our knowledge, this is the youngest case with primary breast non-Hodgkin’s lymphoma.

**Case presentation:**

A 14-year-old Japanese girl felt a lump in her right breast and came to our hospital. A circumscribed soft mass, 30 mm in diameter, was palpable. Histological examination revealed atypical lymphoid cells diffusely spreading into the breast tissue. Based on results of immunohistochemistry and flow cytometry, her disease was diagnosed as B-lymphoblastic lymphoma (stage I). She was then referred to the pediatric department and received combination chemotherapy, based on a chemotherapy regimen for children with acute lymphoblastic leukemia. Following remission induction therapy, we confirmed no FDG uptake in the right breast on PET-CT scan.

**Conclusions:**

We have described a rare malignant lymphoma arising in the breast of an adolescent female. Histological assessment is necessary for diagnosis of breast lymphoma. However, it can be challenging with several reasons, and clinical information may contribute to the assessment. Moreover, treatments for lymphoma vary according to disease types. Thus, surgeons should collaborate closely with pathologists, pediatricians, and hematologists.

## Background

Primary breast lymphoma (PBL) is rare, constituting 0.04–0.53% of malignant breast neoplasms and 1.6% of extranodal lymphoma [1, 2]. However, PBL incidence has recently increased, especially in young women, i.e., those aged younger than 50 [3]. Criteria for the histological diagnosis of PBL were suggested by Wiseman et al [4], and it requires a close association between mammary tissue and lymphomatous infiltration. There should be no evidence of concurrent systemic lymphoma or leukemia, while ipsilateral axillary node involvement does not rule out a PBL diagnosis. It is widely recognized that radiologic findings are generally nonspecific for breast lymphoma [5, 6], probably reflecting a variety of histological patterns with various degrees in infiltration into breast tissue, nodule formation, and/or stromal sclerosis.

PBL is generally B-type, with diffuse large B cell lymphoma (DLBCL) being the most common [7]. Lymphoblastic lymphoma (LBL) is relatively rare, accounting for approximately 2% in non-Hodgkin’s disease cases [7]. The frequency of B cell-LBL (B-LBL) is considered to be around 10–15% in LBL, making the B-LBL in our case extremely rare. Histologically, differentiation between B-LBL and T cell-LBL (T-LBL) is difficult. Tumor cells are frequently arranged in a linear pattern, resembling infiltrating lobular breast carcinoma. The starry-sky pattern is also known to be common in this type of lymphoma. Moreover, tumor cells are reactive for TdT and PAX5, markers of lymphoblastic lymphoma on immunohistochemistry. Meanwhile, the major types of lymphoma in children are Burkitt lymphoma, DLBCL, LBL, and anaplastic large-cell lymphoma, comprising 90% of all non-Hodgkin’s disease cases [8]. Children with LBL are offered the same treatments as patients with acute lymphoblastic leukemia. Usually, combination chemotherapy is effective, and 5-year event-free survival is expected to be more than 90%.

As to malignant breast tumors in children, occurrence rates are reportedly quite low, regardless of tumor types. According to the surveillance, epidemiology and end results (SEER) data, the age-adjusted incidence of breast cancer and sarcoma were 0.03 and 0.06 cases, respectively, per 100,000 females age 19 years and younger [9]. PBL is also extremely rare, and the actual occurrence rate is unknown due to a lack of accumulated data. Furthermore, malignant breast tumors in children might be more likely than breast cancer, sarcoma, or PBL to be metastatic lesions [10]. Rhabdomyosarcoma, neuroblastoma, Ewing sarcoma, melanoma, and renal cell carcinoma are primary tumors reportedly known to be prone to metastasize. Moreover, malignant lymphoma originating from other organs can metastasize to breast tissue and form a mass. Breast lymphomas, representing secondary malignant involvement, are much more frequent than PBL [11]. If a patient has a medical history of these diseases, physicians should take this into account when examining breast tumors.

We herein report a malignant lymphoma of the breast arisen in an adolescent female. To the best of our knowledge, this is the youngest case with primary breast non-Hodgkin’s lymphoma reported to date.

## Case presentation

**Fig. 1:**
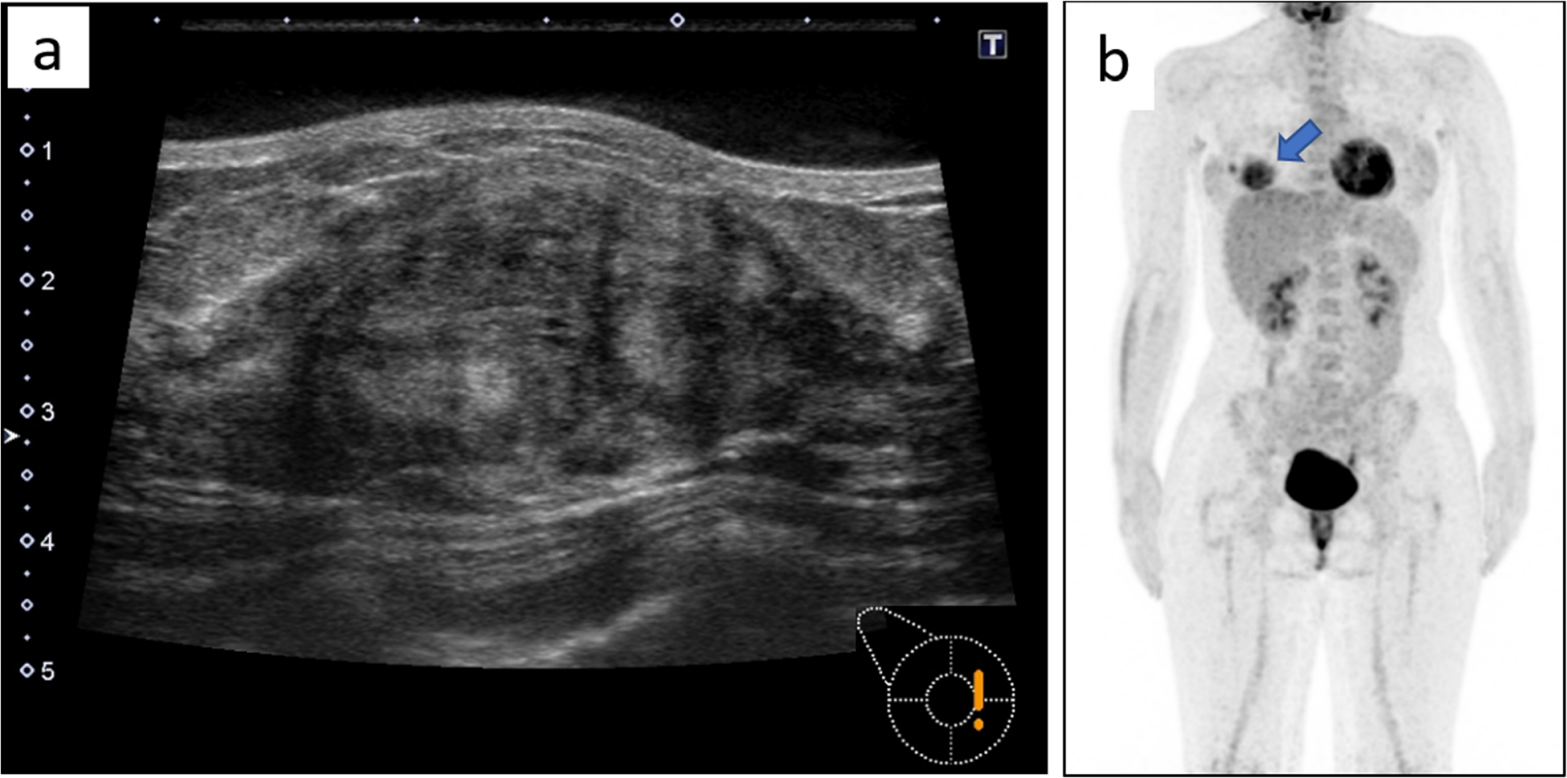
Findings of ultrasound and PET-CT scan. **a** Ultrasonography revealed a 40-mm tumor with an irregular shape. Internal echo was a mixture of low and high levels, suggesting that fibroadenoma, breast cancer, and sarcoma should be included in the differential diagnosis, along with lymphoma. **b** PET-CT scan showed high FDG uptake in the right breast (blue arrow). There were no abnormal hot spots in other organs, including the ipsilateral axilla

A 14-year-old Japanese girl felt a lump in her right breast and came to our hospital. Her medical and family history were unremarkable. A circumscribed soft mass, 30 mm in diameter, was palpable at the internal area of the right breast. There was no swelling of axillary lymph nodes. Ultrasound revealed a 40 mm mass, somewhat irregular in shape, and the internal echo level was a mixture of low and high (Fig. 1a). These findings suggested fibroadenoma, breast cancer, lymphoma, or sarcoma. On PET-CT scan, high FDG uptake (SUVmax 5.05) in the right breast was observed but there were no hot spots in other organs (Fig. 1b).

**Fig. 2:**
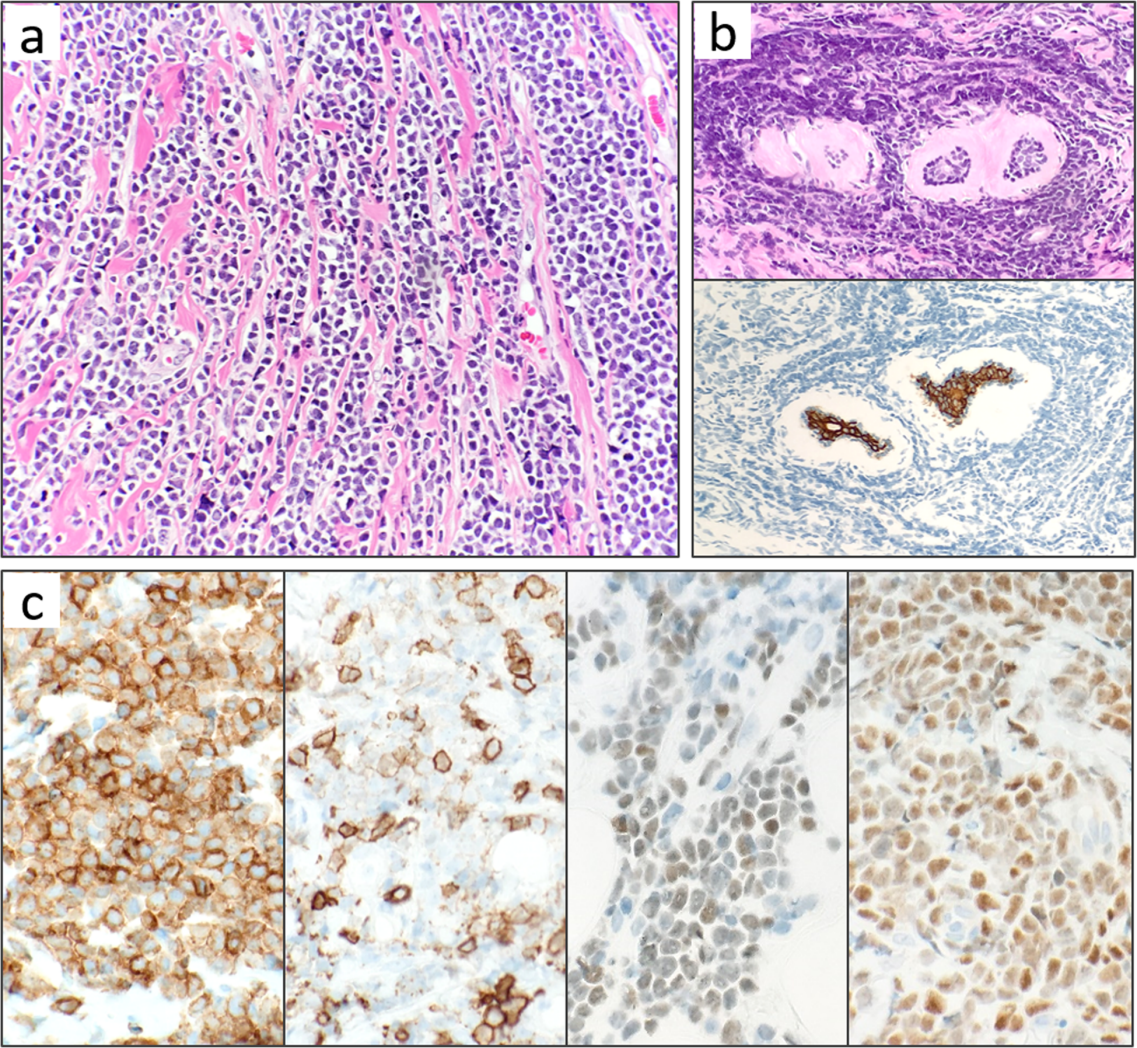
Histopathologic findings of surgical biopsy specimen. **a** Atypical lymphoid cells with irregular nuclear membranes diffusely infiltrate the breast tissue. Cell discohesiveness is apparent, and tumor cells are in a linear pattern, resembling infiltrating lobular breast carcinoma. **b** Periductal infiltration was also observed (upper: hematoxylin and eosin staining). AE1/3 staining (lower), epithelial marker, was confirmed on normal breast ducts. **c** Immunohistochemical findings of CD20, CD10, TdT, and PAX5 (from the left to the right)

Ultrasound-guided needle biopsy was performed, and histologic examination revealed diffuse infiltration of atypical lymphoid cells with irregular nuclear membranes and discohesive features. Malignant lymphoma was suspected but it was difficult to obtain a definitive diagnosis due to crushing artifact of the biopsy sample. Thus, surgical open biopsy was conducted. On microscopic histology, atypical lymphoid cells showed diffuse spreading into the breast tissue (Fig. 2a), and mitotic figures were often observed. Periductal infiltration was also seen (Fig. 2b). Immunohistochemical findings were CD3(−), CD5(−), CD10(+), CD20(+), CD79a(+), TdT(+), PAX5(+), Bcl-2(+), Bcl-6(+), and Ki-67 labeling index (> 80%) (Fig. 2c). Flow cytometry analysis revealed the sample to be positive for CD10, CD19, CD22, and CD45. Rearrangement of J_H_ gene at the immunoglobulin heavy chain locus was confirmed with gene analysis, while T cell receptor beta chain gene rearrangement was negative. Based on these results, we diagnosed B-LBL (stage I). With biopsy from bone marrow, we confirmed that there was no invasion of B-LBL to the bone marrow. She was then referred to the pediatric department and received combination chemotherapy, based on a chemotherapy regimen for children with acute lymphoblastic leukemia. Following remission induction therapy, we confirmed no FDG uptake in the right breast on PET-CT scan. After completion of planned other chemotherapies, she will be followed up without surgery/radiation.

Table 1 lists 8 PBL cases diagnosed at our department in the last 16 years (2004 to 2019). Following the initial diagnosis, all patients were referred to the departments of hematology or pediatrics, as appropriate, and received treatments. One patient (case 6) was transferred to another hospital just after the initial diagnosis, such that the details of her treatments and outcome are unknown. DLBCL was common (6 cases) as widely known. Mean age was 51, while our patient (case 8) was very young. Median survival was 9 years and 3 months, and one patient died due to the lymphoma.

**Table 1:** Eight cases of PBL diagnosed at our department (2004–2019)

Case	Sex	Age	Type	Treatments	Outcome	Overall survival
1	Female	56	Follicular lymphoma	R-CHOP, RT	No recurrence	15 years and3 months
2	Female	34	DLBCL	R-CHOP, MTX	No recurrence	10 years and 2 months
3	Female	45	DLBCL	R-DD-CHOP, MTX	No recurrence	9 years and 5 months
4	Female	65	DLBCL	R-DD-CHOP, HSCT	Recurrence in the contralateral breast	9 years and 3 months
5	Female	39	DLBCL	R-DD-CHOP, MTX, HSCT	No recurrence	7y3mos
6	Female	81	DLBCL	Unknown	Unknown	–
7	Female	72	DLBCL	R-CHOP	Decease due to lymphoma	3 years and 6 months
8	Female	14	B-LBL	ALL-type chemotherapy	No recurrence	8 months

*R-CHOP* rituximab, cyclophospamide, doxorubicin hydrochloride, oncovin, and prednisolone, *RT* radiation therapy, *MTX* methotrexate, *HSCT* autologous peripheral hematopoietic stem cell transplantation, *R-DD-CHOP* dose dense R-CHOP

## Conclusion

We have described a rare malignant lymphoma arising in the breast of an adolescent female. In our case, B-LBL was confirmed to be a very rare PBL presentation, though this is a relatively common lymphoma in children. To the best of our knowledge, the current case is the youngest non-Hodgkin’s PBL case reported to date. Histological assessment is necessary for breast lymphoma facilitating later procedures for obtaining definitive diagnosis and selecting treatments. However, pathological assessment can be challenging. For instance, a small fragmented sample might resemble benign lymphocytic inflammation. Various histological findings according to lymphoma types can also make diagnosis more difficult [11]. Nevertheless, clinical information may contribute to pathological assessment.

Moreover, treatments for lymphoma vary according to disease types. In some types of lymphoma, such as stage 1/2 of mature B cell lymphoma, surgery for the breast lesion may achieve down-staging, allowing chemotherapy doses to be reduced. Generally, surgery is considered not to be beneficial for patients with these tumors [12].

Surgeons should collaborate closely with pathologists, pediatricians, and hematologists.

## Data Availability

Not applicable.

## Abbreviations

B-LBL: B cell lymphoblastic lymphoma
DLBCL: Diffuse large B cell lymphoma
LBL: Lymphoblastic lymphoma
PBL: Primary breast lymphoma
T-LBL: T cell lymphoblastic lymphoma
